# The Potential of Non-Mega Sporting Events for the Promotion of Physical Activity Among Inactive Supporters at the Poznan Half Marathon: A Case Study

**DOI:** 10.3390/ijerph16214193

**Published:** 2019-10-30

**Authors:** Ewa Malchrowicz-Mośko, Joanna Poczta, Katarzyna Adamczewska

**Affiliations:** 1 Faculty of Sport Sciences, Poznan University of Physical Education, 61-871 Poznan, Poland; jpoczta@awf.poznan.pl; 2 Faculty of Health Sciences, Poznan University of Physical Education, 61-871 Poznan, Poland; adamczewska@awf.poznan.pl

**Keywords:** mass run event, sport participation, sport supporters

## Abstract

The impact of sports events on the promotion of physical activity, healthy lifestyles and sports participation is debatable, and most of the literature is on mega-events. This begs the question if more evidence of this type of impact can be found for non-mega events. Research on sports legacy often refers to the tangible effects such as infrastructure that is left after the competition. However, the construction of new facilities does not automatically result in attracting participants. Despite the high expectations of events organizers in terms of their impacts on pro-health behavior of people, few studies provide empirical evidence that events encourage sport fans to become more physically active. The aim of this research was to examine whether a mass run promotes physical activity among spectators, and whether a mass run influences the willingness of spectators to start in half marathon in the future. A written paper–pencil survey was collected from 510 spectators during the 6th Poznan Half Marathon. The results show that observing a mass run event has a positive impact on the willingness to engage in regular physical activity as well as the willingness to take part in this type of sport in the future. Our work provides knowledge about the level of effectiveness in promoting active lifestyles among supporters depending on age, sex and place of residents. This work focuses on mass runs, which have been under-researched when it comes to impact on sport participation.

## 1. Introduction

Sport and physical activity are purported to be important instruments for marketing and promoting healthy lifestyles and tackling health concerns [1]. The ideas of health promotion have gained momentum and are now widely used. Programs at local and international levels are implemented around the world to promote health and a healthy lifestyle. Healthism is a concept that has been part of the political discourse in the 1970s. Crawford defined healthism as a lifestyle that is focused on health and fitness, and based on individual pursuits and building motivation to achieve the assumed health goals. He argued that solving health problems and taking care of health depends on individual determination, selection of skills and overcoming resistance resulting from cultural, advertising, institutional and environmental constraints, work of medical representatives, or simply overcoming laziness and own habits. Healthism also refers to the way in which we approach social and moral values related to health. This process is part of everyday life; it is about how people perceive themselves and the condition of their health against their peers and the social environment. As noted by Crawford, health is not only associated with tensions and dilemmas inscribed not only in the bio-medical domain, but also penetrates the moral, political and social spheres of life [2,3,4,5,6]. Dissemination of physical activity is currently one of the main objectives of programs implemented by international and state authorities (also in Poland), and their aim is to shape pro-health social attitudes. Regular physical activity has multidirectional health benefits, indeed the concentration of activities promoting physical activity is one of the most important public health strategies. Based on Crawford’s work, a number of empirical studies have been undertaken, such as the Cheek’s [7] study on health in regards to advertisements, articles and government initiatives in Australia, Rysst’s study of body ideals and bodily practices in Norway [8], and Greenhalgh’s and Wessely’s research into health in clinical situations in the middle classes of London [9]. As noted by Greenhalgh and Wessely, despite the huge number of publications citing the impact of various social phenomena on health, there is surprisingly little about the perception of this phenomenon by citizens [9]. The impact of organizing sports events on the citizens with-in the context of health promotion, or on those who support participants, has received limited research attention; especially campaigns, which aim to encourage, implement and promote taking up health-oriented sporting activities. These campaigns aim to affect the consciousness of people by influencing the broadly understood relationships between the culture of movement and health. One of such practices is the promotion of widespread sport, especially non-elite mass participation sports events (MPSEs), which may hold potential as a physical activity tool [10]. The vast majority of sporting events include emotions and competition. They may act as motivators, encouraging people to gather in a specific place and time [11,12]. However, little is known about the effectiveness of such activities on health promotion and if it depends on sex, age, place of residence and current levels of physical activity are important determinants in this relationship.

Large scale sporting events are thought to have a positive impact on the physical activity behavior of the events participants [11,12]. The London 2012 bid was based on the promise to use the Olympic Games to promote sports participation for all groups across the UK [13]. There is a perception that mega-events create euphoria amongst supporters, which may translates into motivation and enthusiasm for being active [11,12], but previous evidence shows that spectators of MPSE participants were more satisfied with their experience, and that they held stronger attitudes toward physical activity [14]. Sport participation is a claimed benefit of elite sport events, but the facts do not support that claim [15], and means to capitalize upon events in order to build participation have yet to be developed [16]. Many scholars do not find evidence for this so-called inspiration, demonstration or trickle-down effect from elite sport success to mass sport participation [17,18]. Contrary to what is assumed by the trickle-down effect theorem, successful elite sportspeople do not seem to inspire amateurs to take up sport themselves. In case where studies present evidence that elite sport can have an influence [19], it is more so for those already involved in sport; that is, those who already participate may participate a little more; those who participated in the past, may participate again; or people simply switch from one sport to another, but new participation is not created [15].

While sport and physical activity have long been considered a critical component of the Olympic Games legacy, efforts to evaluate outcomes have proven difficult and methodologies to assess legacy are not standardized across host cities [20]. There is some evidence to support the demonstration effect for those already involved in the sport, but not for new sport participation [21]. Hosting sporting events may offer an opportunity to access scarce resources to create more accessible infrastructure (e.g., sport and recreation facilities and transportation), increase supportive services (i.e., coaching, volunteers and programs), and gain access to specialized equipment [22,23]. However, the construction of new sport facilities will not automatically result enticing users. For example, new facilities were built for the 2004 Summer Olympic Games for sports disciplines, which were not popular among Greeks; these buildings are no longer utilized, remain empty and abandoned [24]. While sports events attract large audiences that gather to support professional athletes, typical recreational events include a huge spread of amateurs and are more closely linked to the person’s behavior and its impact on health. People are motivated to participate in MPSEs for enjoyment, health improvements, stress management, strength and endurance, social interaction, challenge and competition [25,26,27]. One example is running. Watching such an event may potentially encourage spectators or supporters to take on a healthy lifestyle, but may also enforce this through health-enhancing training, which ultimately opens avenues for a successful participation in MPSE. The popularization of running, the growing number of events and contestants taking part in them (also in Poland) encourage to undertake research on the links between organizing running events and the popularization of sport and health-oriented physical activity. This is not only about the general pursuit of restoring the lost physical and mental fitness (classic recreation), but also involves the desire to improve going beyond the boundaries of the status genetically inherited as progressive recreation [28,29]. Not merely active participants, but also viewers who watch an event from outside, can experience self-reflection about their own performance, health and lifestyle. The goal of MPSE may exceed the goals of popularization and include education for active participation in health training.

## 2. The Development of Mass Sport in Poland—Background for Research

In the last few years, the ideology of healthism has been developing in Poland. This trend is particularly visible in mass running events, in which Poles participate eagerly. The scale of this social phenomenon is surely an unusual occurrence on many levels, as a number of interdisciplinary factors should be considered. Physical activity of Poles has increased dynamically in the last two decades. Positive changes began to be observed readily after the political changes in Poland in 1989. Previously, Poles were a society that, unlike Western countries, displayed much lower physical activity. After Poland’s accession to the European Union in 2004, it was clear that Poland was listed towards to lower end of European countries in terms of physical activity. Currently sitting in the middle of the list Poland has seen. Rapid growth, and now the media and politicians are trying to encourage the elderly to engage more in sport [30,31]. Observing older runners during a sporting event can be one option. The social, cultural and economic factors influenced the increase of physical activity for the Poles—and now are better educated, wealthier, have more leisure time and their quality of life has improved. The Poles moved to cities in which sports infrastructure developed dynamically—swimming pools, fitness clubs and bicycle paths. Previously, during communism, they performed a lot of hours of physical labor or agricultural work and had no need for participating in leisurely or lifestyle physical activities such as running or swimming. The end of the 20th century was in a time in Poland of intense and rapid transformation of the planned economy into a free-market capitalist economy. The distance between the Western countries and Poland was at that stage clearly visible, indicators of lifestyle, including sports and recreation, health care, but also, for example, ways of eating. Then Polish society started to adapt to Western lifestyles. Now we observe more positive perception of sport across the Polish society. In the 21st century, Poles perceive sport as an important element of culture and social life. In addition, the ways in which people choose to spend their free time enables people to emphasize their social status. Sport and sport tourism has become a distinctive feature of the middle class in Poland. Especially the fashion for running, not only in their own city, has become very visible [32,33,34,35]. Yet, Poles similarities to the less active sporting inhabitants of the southern European countries like Greece than, for example, to Scandinavia, in which the vast majority of residents are active. The favorite sport pastime of Poles are running (33% of physically active people), swimming (29%) and cycling (53%) [31]. On the other hand, fashion for a healthy and active lifestyle has also become prominent in Poland. For example, running events are no longer solely organized in large cities, but also in smaller Polish towns. People want to look good and be attractive. Among the physically active Poles, the mode of consumption and the forms of physical activity are changing, and heading towards Western patterns. Mass sport and participation in mass sports events are developing dynamically in Poland. 

Bearing in mind the above statements, it was considered important, both from a scientific and utilitarian point of view, to conduct empirical research on the influence of mass-participation running events on willingness to take physical activity among people supporting the participants of the Poznan Half Marathon. The aim of the research was to check whether participation in a mass sports event as a fan motivates to regular physical activity, and whether a mass sports event can influence the willingness to take an active part in this or that kind of running event in the future as a contestant, which is closely related to the desire to undertake systematic, thoughtful physical activity and a change of lifestyle subordinated to the preparations for the participation in the event.

## 3. Materials and Methods 

### 3.1. Design and Aims of the Study

It is often claimed that sport events can stimulate interest and participation in sport. The data in this matter are inconclusive, although the weight of evidence suggests that sport events can sometimes encourage slightly more participation from those who already participate, but do not typically attract new participants [36]. Inclusion in sports, especially with respect to groups that tend to be the least active (e.g. elderly, women and people living in rural areas) is viewed as a key strategy for improving population health. However, the public health reach of such sport events remains an under-researched phenomenon and concerns about the extent of their inclusiveness for all individuals have been raised [37,38]. That is why the aim of this study was to check the level of effectiveness of mass runs in the promotion of active lifestyle among physically inactive supporters depending on socio-demographical variables: age, sex and place of residents (hosts versus sports tourists). Moreover, smaller, non-hallmark events have been, in general, under-researched when it comes to sustainable legacies and their impact on sport participation in particular [21]. The material is empirical research carried out during the 6th Poznan Half Marathon, one of the most important mass runs in Poland. The diagnostic survey method was applied using the on-site standardized interview technique (Question 1: Does participation in the half-marathon as a fan motivates you to undertake regular physical activity (in accordance with the WHO recommendations)? If yes, please indicate the level: 1—very low, 10—very high. Respondents were informed about the WHO recommendations. Question 2: Does cheering in this event have the influence on the level of intensity of the motivation to active participate in the Poznan Half Marathon in your case? If yes, please indicate the level: 1—very low, 10—very high). The sample size was selected in a way that ensures the best possible representativeness of the obtained results. A simple draw scheme without returning was used. While determining the numbers, information from the organizers on the expected number of participants (according to the previous editions) of the event was used. The formula for sample size for finite population was used in the calculations. The assumption was made that the maximum estimation error (e) at 95% confidence level should not exceed 4%. Before the interviews respondents were asked whether they are physically active in accordance with the WHO recommendations (30 min of moderate physical activity for 5 days a week or 20 min of very intense physical activity for 3 days a week). Only people who do not take such behaviors, that is, do not lead regular physical activity and are not active, took part in the study.

### 3.2. Analysis of the Data

Descriptive statistics (percentages, means and standard deviations) were calculated. A chi-square test was used for comparisons of nominal scales, for effect values statistically the value of the effect force (fi) was calculated. In the case of comparisons of the degree of motivation (order Likert scale 1–10), the Mann-Whitney U test was used. The significance level of *α* = 0.05 was assumed, the results were statistically significant when the calculated probability of *p* was satisfied by the inequality of *p* < 0.05. The calculations were performed in the Statistica 10.0 package (StatSoft Inc., Cracow, Poland) from Statsoft Poland.

### 3.3. Participants

The research conducted among the participants of the half-marathon in Poznań did not require the consent of the ethics committee. Questionnaire as a research tool used does not collect sensitive data and identification information about the respondents. Respondents agreed to complete the questionnaire. They did it voluntarily and anonymously. The research was declarative.

Our research shows the socio-demographic profile of supporters of this running event. In total, the survey involved 510 fans, of which 256 were residents of Poznan and the closest neighborhoods, and the remaining 254 fans (sports tourists) were people who declared a different place of residence than Poznan, but the main purpose of their travel was to cheer during the half marathon. The socio-demographic characteristics of respondents are presented below (Table 1). A sample of 510 fans: 180 men respondents and 330 women participated in the research voluntarily. The fans (*n* = 510) were mainly 19–25 (40.4%—211) and 26–35 years old (27.8%—142). Among the surveyed people, fans with secondary education (32.3%—164) and with higher education (31.8%—162) constituted the vast majority. A greater percentage of them—50.6% (258) were professionally active, over 31.8% were students (162).

## 4. Test Results 

Almost three-quarters of the fans—as many as 73.7% (376)—stated that participation in the half-marathon as a fan motivates them to undertake regular physical activity (in accordance with the WHO recommendations). A quarter of respondents (26.3%) had a different opinion on the subject, they did not feel motivated to undertake physical activity due to observing this sporting event.

In order to further analyze the obtained result, it was decided that people who responded positively (*n* = 376) should be asked to define the intensity level of this motivation on a scale of 1 to 10 points on a 10-point Likert scale. The result of the study turned out to be equally high. It turned out that as many as 27.7% of respondents declaring motivation to work on their own physical activity determined its intensity at the level of 10 points. The respondents felt motivated to undertake regular physical activity (PA) thanks to cheering in the half marathon on average at the level of 7.8 points (Table 2).

More than half of the fans (53.9%—275) felt motivated to take part in the half marathon in the future as a result of being a fan during this event, while 46.1% did not. The degree of intensity of this motivation turned out to be high. Supporters of the 6th Poznan Half marathon felt motivated to actively participate in this event in the future as competitors with an average of 7.5 points, as shown in Table 3.

In the next step of the research procedure, it has been interesting to note that if the participation in the half-marathon varied as a fan motivator to undertake physical activity depending on the sex, age or place of residence (locals vs. sports tourists).

Table 4 presents the motivations for PA between the surveyed men and women.

Almost three-quarters of the female fans—as many as 72.42%—and 76.11% of surveyed men fans stated that participation in the half-marathon as a fan motivates them to undertake physical activity. The level of intensity (according to Likert scale) was on the high level of 7.82 for women and 7.87 for men (Table 5).

Surveyed fans were also asked if the cheering in the surveyed event have an influence on the level of intensity of the motivation to participate in the Poznan Half Marathon in the future (Table 6.) Almost half of the women fans—as many as 52.12%—and 57.22% of surveyed men fans stated that participation in the half-marathon as a supporter motivates them to participate in this event in the future.

The level of intensity (according to Likert scale) was on the high level of 7.39 for women and 7.62 for men (Table 7). 

Differences between two groups of respondents were not found (*p* > 0.05). Men and women felt similarly motivated in a positive way during a half marathon.

Further analysis of the results showed that 70.70% of the residents and 76.77% of sports tourists felt motivated to undertake regular PA and to participate in the half marathon in the future as a result of cheering during this event (Table 8). The level of the intensity of this motivation presents Table 9 (high level: tourists 7.93 and residents: 7.73).

Differences between two groups of respondents were not found (*p* > 0.05).

Table 10 presents the study of the level of intensity of the motivation to participate in the Poznan Half Marathon in the future—depending on the place of the residence—were on the average level between locals—50.56% and 56.30% between tourists. The level of intensity of the motivation to participate in the Poznan Half Marathon in the future—depending on the place of the residence (Table 11)—was at the average level 7.5 for sports tourists and 7.45 for residents of Poznan.

Differences between two groups of respondents were not found (*p* > 0.05). Sports tourists and hosts felt similarly motivated.

Young and older surveyed fans were also asked if the cheering in the surveyed event have the influence on the level of intensity of the motivation to undertake PA due to cheering in the 6th Poznan Half Marathon. They were divided (Table 12) according to age. Almost 71.08% people at the age up to 25 years old, and 75% of people aged over 50 years old answered yes.

The level of intensity of the motivation to undertake PA due to cheering in the 6th Poznan Half Marathon—depending on age (according to Likert scale)—was on the high level of 7.86 for respondents at the age up to 25 years old and 8.17 for respondents at the age over 50 years old—Table 13 (8.17 was the highest result).

Differences between two groups of respondents were not found (*p* > 0.05). Young and older surveyed fans were asked if the cheering in the surveyed event have the influence on the level of intensity of the motivation to participate in the Poznan Half Marathon in the future (Table 14). “Yes” said 56.63% people at the age up to 25 years old, and 37.50% people at the age over 50 years old.

The statistically significant difference (*p* < 0.05) between these two groups of respondents was found. Significant (*p* = 0.0150) with a small effect (*fi* = 0.14).

A similar percentage of older and younger people felt encouraged to take regular physical activity, however, people under 25 years old statistically more often felt motivated to start in the running event in the future than people over 50 years old.

The level of intensity of the motivation to participate in the Poznan Half Marathon in the future—depending on age (according to Likert scale)—was on the high level of 7.50 for respondents at the age up to 25 years old and 7.72 for respondents at the age over 50 years old (Table 15).

Differences between two groups of respondents were not found (*p* > 0.05).

## 5. Discussion

Respondents were divided into three groups depending on sex, place of residence and age. Almost three-quarters of surveyed fans—as many as 73.7% (376)—stated that participation in the half-marathon as a fan motivates them to undertake regular physical activity. More than half of the fans (53.9%—275) felt motivated to take part in the half marathon in the future as a result of being a fan during this event. Respondents, declaring motivation to work on their own physical activity determined its intensity at the level of 10 points of the Likert scale, and felt motivated to undertake physical activity due to cheering in the half marathon on average at the level of 7.8 points; felt motivated to actively participate in this event in the future as the competitors with an average of 7.5 points. The intensity of the motivation to undertake PA due to cheering in the 6th Poznan Half Marathon depending on sex: women—72.42 % (Likert scale 7.82); men—76.11% (Likert scale 7.87); place of residence: residents—70.70% (Likert scale 7.73); sports tourists—76.77% (Likert scale 7.93); age up to 25 years old—71.08% (Likert scale 7.86) and over 50 years old—75% (Likert scale 8.17). The intensity of the motivation to participate in the Poznan Half Marathon in the future due to cheering in this event depending on: sex: women—52.12% (Likert scale 7.39); men—57.22% (Likert scale 7.62); place of residence: residents—50.56% (Likert scale 7.45); sports tourists—56.30% (Likert scale 7.5); age up to 25 years old—56.63% (Likert scale 7.5) and 50—37.5% (Likert scale 7.72) were significant (*p* = 0.0150) with a small effect (*fi* = 0.14).

As a result, it might be concluded that the half marathon encouraged physical activity regardless of age, gender or place of residence (citizens of host city vs. sports tourists). This is important information as it turned out that the organization of non-mega sporting events such as mass running events had a potential positive impact on physical activity of their inactive fans (also among women, older people and citizens of host city). Over 70% of the surveyed fans were ready to start regular physical activity and over 50% were ready to start in the mass run in the future.

Young people were statistically more motivated than older people to take part in a running event in the future. It is a challenge for event organizers to find a way to encourage also older people to become runners. If it could be expected that young people would be more motivated to participate in a running event in the future (ant test result confirmed it), it could have been the same that young people would be more motivated to take up physical activity. Meanwhile, older people felt equally motivated to do physical activity. This is important information from a scientific and utilitarian point of view—sport managers should therefore try to include the widest possible range of older people as supporters in the event so that they can feel the desire to do physical activity.

Previous studies present evidence that elite sport can have an influence [19], but it was more so for those already involved in sport; that is, those who already participate may participate a little more; those who participated in the past, may participate again; or people simply switch from one sport to another, but new participation is not created [15]. The results of our research show that new participation (among inactive physically people) could be created by supporting during a mass sport event.

## 6. Final Conclusions

Running as one of the forms of physical activity that does not require large financial outlays, especially at the beginning. It is a sport for all that can be taken practically anywhere. You can run recreationally or take on the challenge of taking part in numerous running events. Then the appropriate process of preparing the body for participation in the long-distance runs begins to have a huge significance. Systematic work, on the other hand, has beneficial effects both in the physical and mental spheres [39]. For this reason, the results of the conducted research are potentially optimistic. They indicate that the organization of mass sports events like the half marathon motivates people to undertake physical activity and, to a large extent, encourages them to actively participate in these events in the future (according to respondents declarations). The research shows that the half marathon also encourages the elderly persons to physical activity. However, physical activity adapted to different age categories should also be promoted, and event organizers, apart from directing their attention to sponsors, city promotion or tourism development through sport events, should also take into account the promotion of safe physical activity, preferably under the supervision of doctors and trainers, not on their own, especially among older age categories.

The strength of our study was the high sample size of our respondents. The limitations were the declarative character of our study. We did not know if respondents’ intentions to participate would be realized in real life. The future study that will examine real sport participation will be beneficial. In the future we will also involve more characteristics of respondents in our analyses, e.g., their connections with the relatives who are practicing sport regularly.

## Figures and Tables

**Table 1:** Socio-demographic characteristics of respondents.

Socio-Demographic Characteristics	All (*n* = 510)	
	*n*	%
Sex		
Men	180	35.3
Women	330	64.7
Age		
<18	38	7.5
19–25	211	41.4
26–35	142	27.8
36–50	71	13.9
51–70	46	9.0
71>	2	0.4
Education level		
Primary education	30	5.9
Vocational education	14	2.7
Secondary education	164	32.2
Incomplete higher education	75	14.7
Completed higher education	162	31.8
Employment status		
School pupil (<18 years)	51	10.0
Student	162	31.8
Professionally active	258	50.6
Unemployed	25	4.9
Pensioner	14	2.7
Place of residence		
village	88	17.3
City up to 10,000 inhabitants	63	12.4
City 10–100,000 inhabitants	82	16.1
City 100–500,000 inhabitants	32	6.3
Locals/Sports tourists		
Locals	256	50.2
Tourists	254	49.8

**Table 2:** Study of the level of intensity of the motivation to undertake physical activity (PA) due to cheering in the 6th Poznan Half Marathon.

Mean	Median	Minimum	Maximum	Standard Deviation
7.8	8	1	10	1.9

**Table 3:** Study of the level of intensity of the motivation to participate in the future editions of Poznan Half Marathon.

Mean	Median	Minimum	Maximum	Standard Deviation
7.5	8	1	10	2.0

**Table 4:** Study about the motivation to undertake PA due to cheering in the 6th Poznan Half Marathon—depending on sex.

Reply	Women		Men		χ²	*p*
	*n*	%	*n*	%		
Yes	239	72.42%	137	76.11%	0.82	0.3660
No	91	27.58%	43	23.89%		
Altogether	330	100.00%	180	100.00%		

**Table 5:** The level of intensity of the motivation to undertake PA due to cheering in the 6th Poznan Half Marathon—depending on sex.

Sex	*n*	Mean	Median	Standard Deviation	Z	*p*
Women	239	7.82	8	1.91	0.35	0.7196
Men	137	7.87	8	1.92		

**Table 6:** Study about the motivation to participate in the Poznan Half Marathon in the future—depending on sex.

Reply	Women		Men		χ²	*p*
	*n*	%	*n*	%		
Yes	172	52.12%	103	57.22%	1.22	0.2694
No	158	47.88%	77	42.78%		
Altogether	330	100.00%	180	100.00%		

**Table 7:** The level of intensity of the motivation to participate in the Poznan Half Marathon in the future—depending on sex.

Sex	*n*	Mean	Median	Standard Deviation	Z	*p*
Women	172	7.39	8	2.09	0.95	0.3338
Men	103	7.62	8	1.98		

**Table 8:** Study about the motivation to undertake PA due to cheering in the 6th Poznan Half Marathon—depending on the place of the residence.

Reply	Residents		Sports Tourists		χ²	*p*
	*n*	%	*n*	%		
Yes	181	70.70%	195	76.77%	2.42	0.1195
No	75	29.30%	59	23.23%		
Altogether	256	100.00%	254	100.00%		

**Table 9:** The level of intensity of the motivation to undertake PA due to cheering in the 6th Poznan Half Marathon—depending on the place of the residence.

Place of the Residence	*n*	Mean	Median	Standard Deviation	Z	*p*
Sports Tourists	195	7.93	8	1.96	1.38	0.1685
Locals	181	7.73	8	1.85		

**Table 10:** Study about the motivation to participate in the Poznan Half Marathon in the future—depending on the place of the residence.

Reply	Locals		Sports Tourists		χ²	*p*
	*n*	%	n	%		
Yes	132	51.56%	143	56.30%	1.15	0.2833
No	124	48.44%	111	43.70%		
Altogether	256	100.00%	254	100.00%		

**Table 11:** The level of intensity of the motivation to participate in the Poznan Half Marathon in the future—depending on the place of the residence.

Group	*n*	Mean	Median	Standard Deviation	Z	*p*
Sports Tourists	143	7.50	8	2.07	0.23	0.8170
Locals	132	7.45	8	2.02		

**Table 12:** Study about the motivation to undertake PA due to cheering in the 6th Poznan Half Marathon—depending on age.

Reply	Age up to 25 Years Old		Age over 50 Years Old		χ²	*p*
	*n*	%	*n*	%		
Yes	177	71.08%	36	75.00%	0.30	0.5813
No	72	28.92%	12	25.00%		
Altogether	249	100.00%	48	100.00%		

**Table 13:** The level of intensity of the motivation to undertake PA due to cheering in the 6th Poznan Half Marathon—depending on age.

Age	*n*	Mean	Median	Standard Deviation	Z	*p*
Age up to 25 years old	177	7.86	8	1.90	−0.95	0.3440
Age over 50 years old	36	8.17	8	1.86		

**Table 14:** Study about the motivation to participate in the Poznan Half Marathon in the future—depending on age.

Reply	Age up to 25 Years Old		Age over 50 Years Old		χ²	*p*	fi
	*n*	%	*n*	%			
Yes	141	56.63%	18	37.50%	5.92	0.0150	0.14
No	108	43.37%	30	62.50%			
Altogether	249	100.00%	48	100.00%			

**Table 15:** The level of intensity of the motivation to participate in the Poznan Half Marathon in the future—depending on age.

Age	*n*	Mean	Median	Standard Deviation	Z	*p*
Age up to 25 years old	141	7.50	8	1.93	−0.88	0.3785
Age over 50 years old	18	7.72	8	2.44		
